# Supplementary material for: The Evolution and Characterization of the RNA Interference Pathways in Lophotrochozoa
Source: Genome Biol Evol. 2024 May 7;16(5):evae098. doi: 10.1093/gbe/evae098 (PMC11114477; doi:10.1093/gbe/evae098)
Supplement: evae098_Supplementary_Data [file evae098_supplementary_data.zip › Supplementary_materials.zip!/selected_miRNAs/NoGe_selected_miRNAs/known_miRNA_families_pdf_files/NMRB01000380.1_Notospermus_geniculatus_strain_Ushimado_scaffold380,_whole_genome_shotgun_sequence_11928.pdf]

Provisional ID	:	NMRB01000380.1_Notospermus_geniculatus_strain_Ushimado_scaffold380,_whole_genome_shotgun_sequence_11928
Score total	:	200834.2
Score for star read(s)	:	3.9
Score for read counts	:	200825.3
Score for mfe	:	2
Score for randfold	:	0
Score for cons. seed	:	3
Total read count	:	393922
Mature read count	:	393541
Loop read count	:	0
Star read count	:	381

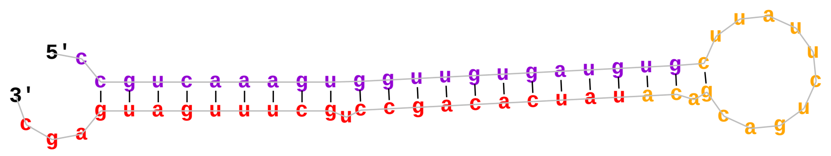

Genome coordinates: NMRB01000380.1 235191-235251

pre-miRNA sequence:
CCGUCAAAGUGGUUGUGAUGUGCCGUCAAAGUGGUUGUGAUGUGCUUAUUCUGACGACAUAUCACAGCCUGCUUUGAUGAGC

Mature sequence: UAUCACAGCCUGCUUUGAUGAGC

Aligned mature sequence of the *N. geniculatus* pre-miRNA with the top 5 hits from the BLAST search against the pre-miRNA mirGeneDB set:

```
                            ********** ***********
Nge_Mir-2-P4i               uaucacagccugcuuugaugagc
Lan-Mir-2-o78_3p            uaucacagccugcuuugaugagc
Lgi-Mir-2-o15_3p            uaucacagccagcuuugaugagu
Npo-Mir-2-o24_3p            uaucacagccugcuuugaugaga
Lan-Mir-2-o71_3p            uaucacagccugcuuugaugagc
Lan-Mir-2-o73_3p            uaucacagccugcuuugaugaga
```
